# Unravelling molecular mechanisms involved in resistance priming against downy mildew (*Plasmopara viticola*) in grapevine (*Vitis vinifera* L.)

**DOI:** 10.1038/s41598-023-41981-x

**Published:** 2023-09-06

**Authors:** Nicolas Vigneron, Jérôme Grimplet, Eric Remolif, Markus Rienth

**Affiliations:** 1 University of Sciences and Art Western Switzerland, Changins College for Viticulture and Enology, Route de Duillier 60, 1260 Nyon, Switzerland; 2 Departamento de Ciencia Vegetal, Centro de Investigación y Tecnología Agroalimentaria de Aragón, Avda. Montanaña 930, 50059 Zaragoza, Spain; 3 Instituto Agroalimentario de Aragón-IA2 (CITA-Universidad de Zaragoza), 50013 Zaragoza, Spain; 4 Agroscope, Plant Protection, Mycology, Route de Duillier 60, 1260 Nyon, Switzerland

**Keywords:** Transcriptomics, Plant molecular biology, Effectors in plant pathology

## Abstract

Downy mildew (DM; *Plasmopara viticola*) is amongst the most severe fungal diseases in viticulture and the reason for the majority of fungicide applications. To reduce synthetic and copper-based fungicides, there is an urgent need for natural alternatives, which are being increasingly tested by the industry and the research community. However, their mode of action remains unclear. Therefore, our study aimed to investigate the transcriptomic changes induced by oregano essential oil vapour (OEOV) in DM-infected grapevines. OEOV was applied at different time points before and after DM infection to differentiate between a priming effect and a direct effect. Both pre-DM treatment with OEOV and post-infection treatment resulted in a significant reduction in DM sporulation. RNA-seq, followed by differential gene expression and weighted gene co-expression network analysis, identified co-expressed gene modules associated with secondary metabolism, pathogen recognition and response. Surprisingly, the molecular mechanisms underlying the efficiency of OEOV against DM appear to be independent of stilbene synthesis, and instead involve genes from a putative signalling pathway that has yet to be characterized. This study enhances our understanding of the molecular regulation of innate plant immunity and provides new insights into the mode of action of alternative natural antifungal agents.

## Introduction

Grapevine (*Vitis vinifera* L.) is economically among the most important perennial fruit crops in the world, with a total of 7.3 mha having been used for grape production in 2022^1^. *Vitis vinifera* L*.* is highly susceptible to various fungal diseases, such as downy mildew (DM), powdery mildew and botrytis bunch rot, which are responsible for important losses in yield and quality within the vitivinicultural industry. To control these pathogens, large amounts of pesticides are required and applied every year, questioning the sustainability of viticulture from a producers, consumers and environmental point of view^2^. One of the most devastating grapevine diseases is downy mildew, which is caused by the oomycete *Plasmopara viticola* (*P. viticola*, Berk. & M. A. Curtis; Berl. & De Toni)*.* DM infections can strongly impact grapevine physiology, leading to decreases in yield and a deterioration in the organoleptic quality of the resulting wines^3,4^. *Plasmopara viticola* infections occur primarily on leaves and can result in large losses in photosynthetic leaf area. The pre-véraison green berry can also become infected, leading to the subsequent withering of the berry at later stages. In leaves, *P. viticola* enters through the stomata and intracellularly colonizes the plant tissue. After a period of incubation inside the leaves, the sporangia-carrying sporangiophore exit the leaf via the stomata. The released zoospores will colonize further leaves or other plants, causing secondary infections. *Plasmopara viticola* can also reproduce via sexual reproduction, in which so-called oospores overwinter in leaf debris and release macrosporangia the next spring; these will then release zoospores and restart the infection cycle^4^.

Various strategies exist to control DM in the vineyard. The application of synthetic fungicides is still one of the most effective and common strategies, but it has obvious negative side effects that impact the environment^5^ and consumer and producers’ health^2^. It can also lead to the development of pathogen resistance, such as that described for the class of QoI pesticides^6^. To date, the only effective organic solution for controlling DM in the vineyard is the application of copper-based fungicides, which has the drawback of leading to copper accumulation in soils^5^; furthermore these fungicides require a higher frequency of application than synthetic ones due to their low rain fastness.

In the pursuit of decreasing the environmental impact of viticulture, meeting the growing demand from consumers and growers for organic products with little or no harmful residues in grapes, wines and the environment, other alternative solutions are constantly being explored. These include, among others, the application of natural substances, such as sage extracts^7^, chitosan^8^, other diverse plant extracts, different bacteria^9^, dsRNA^10^, sulfated laminarin/PS3^11,12^, BTH (Benzothiadiazole), methyl jasmonate, PHOS (phosphonates)^13^, peptide NoPv1^14^, BABA (β-aminobutyric acid)^15^, kaolin^16^, d-Tagatose^17^, Trichoderma harzianum^18^ or Cerevisane^19^. Essential oils from the Lamiaceae family^20^, such as *Origanum vulgare,* is a particularly promising natural product whose direct action on numerous pathogens has already been demonstrated^20–22^. It has also been found to be very efficient in trials conducted in climate chambers on grapevine^23,24^.

Many of these natural substances have shown high efficiency against *P. viticola* under certain laboratory and well-controlled conditions. However, the results of field trials are often extremely variable and not repeatable, and are highly dependent on the climatic conditions that prevail during the growing season. It is mostly not clear to what extent the effect of these substances on pathogen development is direct or via a stimulation or priming of the innate immune system of the plant. Priming improves the defensive capacity of plants upon stimulus perception and can occur in the plant at the physiological, transcriptional, metabolic and epigenetic levels^25^.

This is a crucial aspect to consider to ensure the optimization of the application strategy for natural substances and the development of new natural biopesticides.

Few studies have been conducted to elucidate the molecular mechanisms of these alternative substances using transcriptomic and/or metabolomic approaches^13,17,23,26^. According to the available published studies, the majority of these alternative solutions seem to enhance plant defense, leading to better control of the disease, either through pre-activation of the signaling cascade or due to the faster onset of phytoalexin biosynthesis^27^.

Several molecular studies have provided deeper insight into the *Vitis*—*P. viticola* pathosystem of resistant and non-resistant cultivars^11,28–33^; nevertheless, the molecular mechanisms of plant priming or the elicitation of the vine innate immunity are still poorly understood and characterized. The present experiments and bioinformatic analysis of published data aimed, therefore, to obtain a deeper insight in the role of transcriptomic modulation induced by natural substances.

## Results

### Effects on sporulation

**Figure 1:**
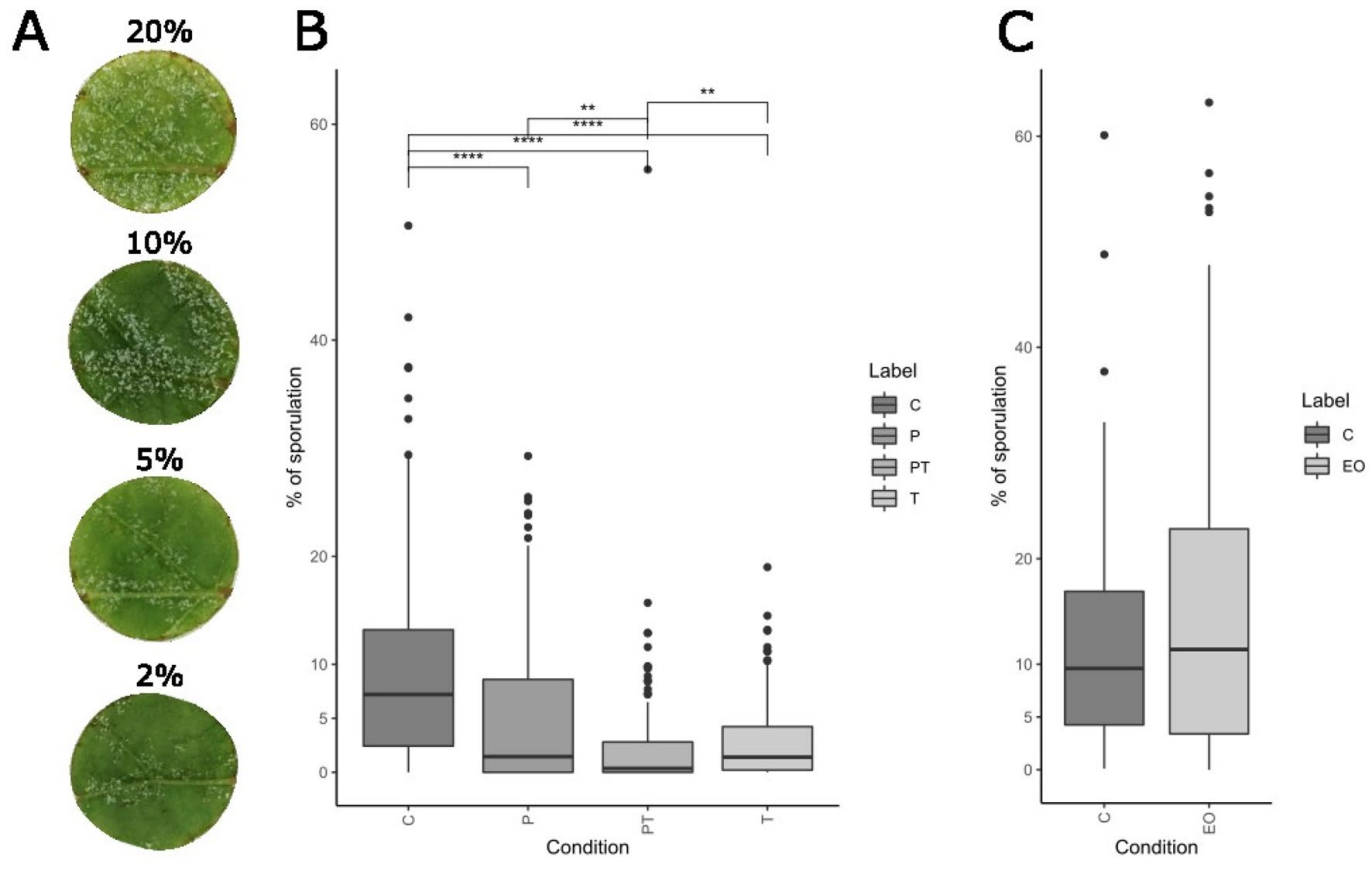
Effect of essential oil on downy mildew infection. (**A**) Results of the image analysis method. (**B**) Preventive effect of essential oil at 2.5 µL/L of air in different conditions. P, PT and T represents primed conditions, primed treated conditions, and treated conditions respectively. (**C**) Curative effect of essential at 2.5 µL/L of air. EO represents the EO-treated sporangia before inoculation.

The appearance of so-called oil spots is the first visible sign of *P. viticola* infection and occurs after an incubation period of 5–10 days after infection. Subsequently, sporulation on the abaxial side of the leaf can be triggered by keeping it at 100% humidity*.* DM sporulation is usually assessed by visible observation using the OIV452 descriptor. This method has been successfully developed for the field screening of OIV 2009^34^. However, in laboratory conditions, this descriptor lacks precision and objectivity and is thus highly variable depending on the observer. Several simple methods have been developed to increase the objectivity, precision and reproducibility of DM infection assessment^35,36^. Peressotti et al.^35^ developed a semi-automatic method using an open-source software, imageJ, to quantify DM infection in leaf discs assays. We adopted this method to assess DM infection, which is expressed as the percentage of leaf surface covered by spores (Fig. 1A). While this method is accurate, objective and reproducible, the obtained percentage of sporulation is 2–5 times lower than that in assessments using classic visual methods.

Oregano essential oil vapor (OEOV) has been shown to be effective against DM infection in climate chamber experiments^23^, but effective concentration ranges and the timing of application, as well as the underlying molecular mechanisms, remain to be further elucidated. We therefore decided to perform a series of experiments with different treatment conditions to better understand the molecular mode of action of OEOV and to discriminate between plant defense enhancement and/or a putative curative action.

In our leaf disc system, we tested four different concentrations (10, 5, 3.75 and 2.5 µL OEOV/L of air), and observed a concentration-dependent effect (Supplementary Fig. 1). Due to the phytotoxicity of the OEOV at high concentrations, the two lowest concentrations were the most efficient (low to no phytotoxicity and good disease control) and drastically reduced DM sporulation regardless of treatment conditions (Fig. 1B). Both the primed (P; 24 h pre-inoculation) and treated (T; just after inoculation) conditions reduced sporulation fivefold compared to the control. When priming and treatment were combined (PT), the sporulation was reduced by a factor of 20 (Fig. 1B). The OEOV also seemed to exhibit a certain preventive effect (Fig. 1B). To test the hypothesis that OEOV has a direct curative effect, we exposed the leaf discs showing visible downy mildew spores to OEOV for 1 h to avoid any indirect reaction resulting from plant defense, then harvested the spores and inoculated new, healthy leaf disks with this inoculum. No difference in subsequent sporulation between the control and the OEOV-treated conditions was observed, thus questioning the direct curativeeffect of OEOV on DM spores (Fig. 1C). The main effect of OEOV seems to be indirect and might thus occur via an enhanced plant-defense response which limits pathogen development. To better understand this potential enhancement of plant defense, we performed RNA-seq of leaf discs at three different time points (24 hpi, 48 hpi and 72 hpi) to screen for differentially expressed genes.

### RNA-seq analysis

**Figure 2:**
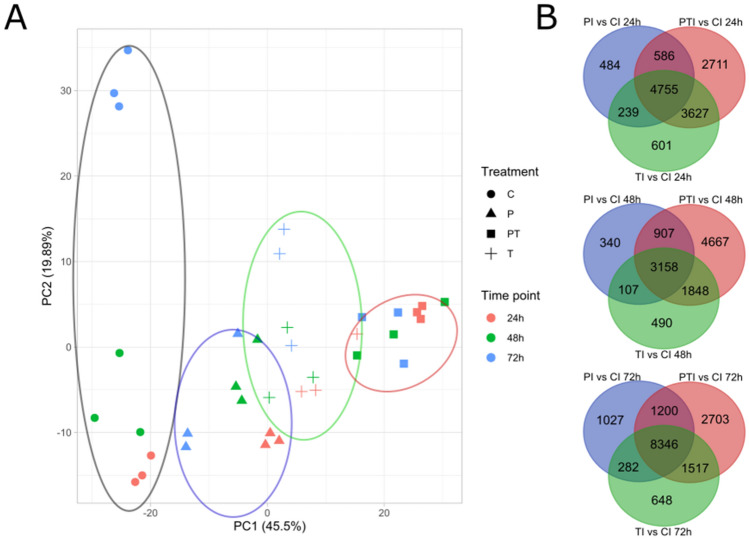
Regulatory effect of OEOV treatment on *V. vinifera cv Chasselas* transcriptome during *P. viticola* infection. (**A**) Principal component analysis on transformed read counts. (**B**) Venn diagram of DEGs.

The PCA on the full expression dataset shows a clear separation based on treatment conditions, with the inoculated control being totally separated from the treated samples (Fig. 2A), and PT also being separated from the other two treatment conditions. Numerous DEGs were shared among treatment conditions at the different time points (4755 at 24 hpi, 3158 at 48 hpi and 8346 at 72 hpi) (Fig. 2B). PT and T had most DEGs in common at every time point (3627 at 24 hpi, 1848 at 48 hpi, 1517 at 72 hpi) and P and T shared the least DEGs at each time point (239 at 24 hpi, 107 at 48 hpi, 282 at 72 hpi). This highlights the transient effect of OEOV on potential plant defense enhancement.

### Weighed co-expression genes network analysis (WGCNA)

To better understand the molecular mechanism of OEOV in the DM/grapevine pathosystem in combination with the treatments, a WGCNA was performed on the expression data to identify co-expressed genes (Supplementary Fig. 2A). Modules produced by the WGCNA were correlated with the median of sporulation for each condition. By correlating modules and the percentage of sporulation, it is possible to obtain genes linked to infection. The more these are positively correlated, the more they should facilitate infection and subsequent sporulation. If the module is negatively correlated, they should be involved in resistance and act against the pathogen’s infection and subsequent sporulation. Twelve modules were identified, with four being highly correlated to sporulation. Black and tan modules were negatively correlated to sporulation (− 0.78 and − 0.96 respectively) and green-yellow and turquoise modules were positively correlated to sporulation (0.81 and 0.74 respectively) (Supplementary Fig. 2B). These four highly correlated modules were selected for further analysis. Module membership (MM), which corresponds to a correlation between the module Eigengenes and gene expression, was subsequently calculated. We then filtered genes with an MM above an absolute 0.8 threshold for each selected module to reduce module noisiness and thereby retain only strongly co-expressed genes. Gene numbers per module were 247 for the black module, 3 for the tan module, 301 for the green-yellow module and 2901 for the turquoise module.

### Gene ontology enrichment

**Figure 3:**
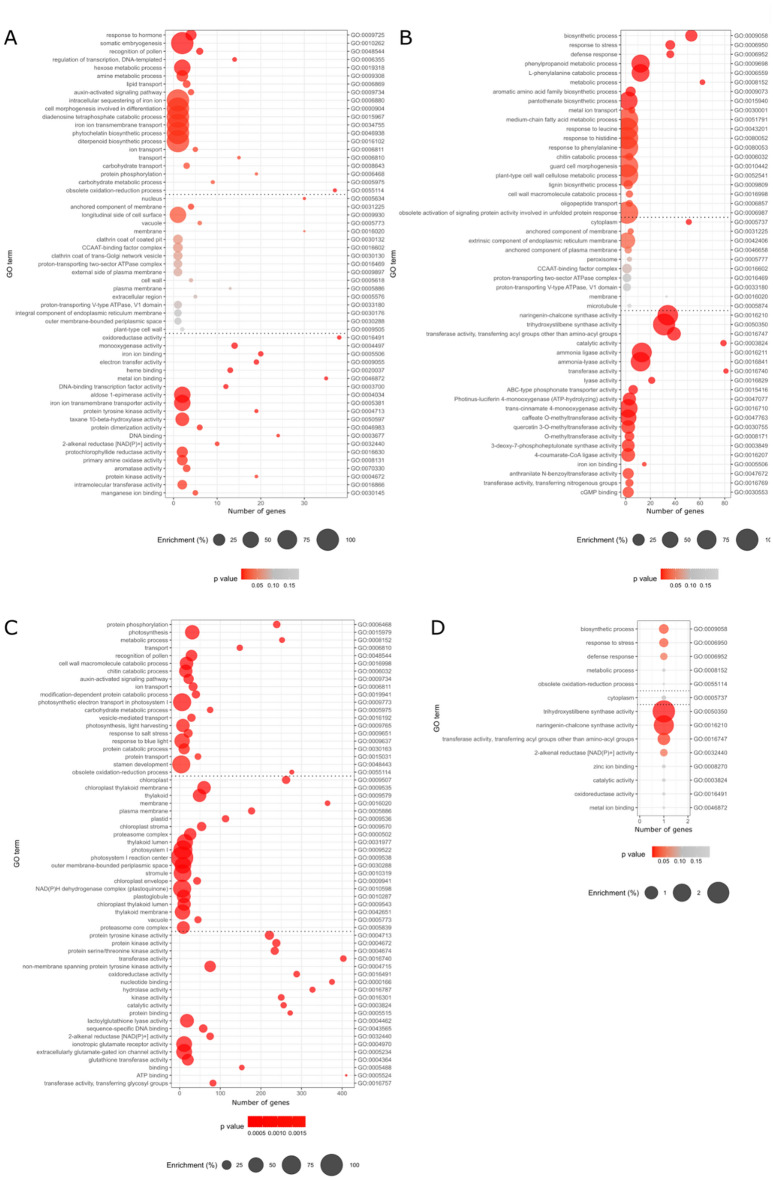
Gene ontology for genes present in WGCNA top modules. (**A**) Genes present in negatively correlated black module. (**B**) Genes present in positively correlated green-yellow module. (**C**) Genes present in positively correlated turquoise module. (**D**) Genes in negatively correlated tan module.

To investigate the potential common function or pathway of each module, GO enrichment was performed (Fig. 3).

For the black module (Fig. 3A), only three GO terms were strongly enriched: somatic embryogenesis (GO:0010262), longitudinal side of cell surface (GO:0009930) and response to hormones (GO:0009725). Four GOs had a high number of genes: oxidoreductase activity (GO:0016491) with 38 genes enriched, obsolete oxidation–reduction process (GO:0055114) with 37 genes enriched, then nucleus (GO:0005634) and membrane (GO:0016020) with both 30 genes enriched. For the green-yellow module (Fig. 3B), 8 GO terms were strongly enriched: trihydroxystilbene synthase activity (GO:0050350), naringenin-chalcone synthase activity (GO:0016210), ammonia ligase activity (GO:0016211), phenylpropanoid metabolic process (GO:0009698), l-phenylalanine catabolic process (GO:0006559), extrinsic component of endoplasmic reticulum membrane (GO:0042406), acyltransferase activity, transferring groups other than amino-acyl groups (GO:0016747), biosynthetic process (GO:0009058). For the turquoise module (Fig. 3C), 8 GO terms were strongly enriched with trihydroxystilbene synthase activity (GO:0050350), photosynthesis (GO:0015979), chloroplast thylakoid membrane (GO:0009535), thylakoid (GO:0009579), non-membrane spanning protein tyrosine kinase activity (GO:0004715), protein tyrosine kinase activity (GO:0004713), protein kinase activity (GO:0004672), chloroplast (GO:0009507), protein serine/threonine kinase activity (GO:0004674). For the tan module (Fig. 3D), no significant enrichment was observed due to the very small size of the module; the top GO terms were trihydroxystilbene synthase activity (GO:0050350) and naringenin-chalcone synthase activity (GO:0016210) with enrichment at 3.1 and 2.4%.

### Co-expression gene network analysis

**Figure 4:**
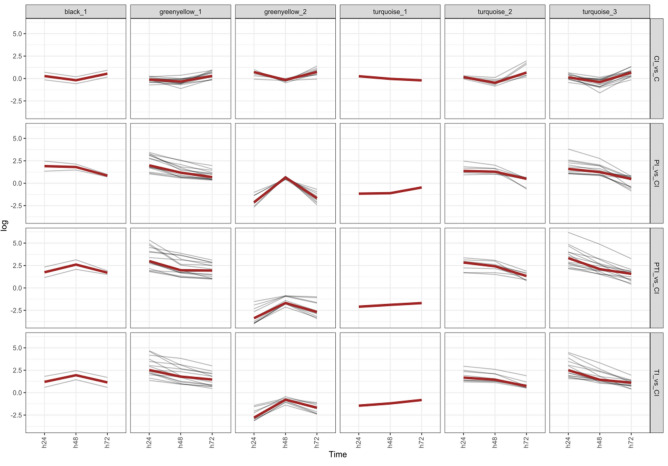
Transcriptional response of differential expressed genes (DEGs) distributed among the top modules. Expression of DEGs with p < 0.05 and abs(log) > 1.

To better characterize the induced regulations involved in resistance induction, a second clustering algorithm (*clust)* was applied in combination with a DEG analysis to clean WCGNA clusters^37^ of any outliers and help identify highly specific co-expressed genes (Fig. 4). This algorithm yields very specific submodules, eliminating numerous genes per module and/or creating submodules with tightly regulated genes. For the black module, 2 out of the 247 genes were selected: a G-type lectin S-receptor kinase (Vitvi19g01964) and a LRR RLK (Vitvi08g01352) (Fig. 4, Supplementary Table 1). Both of these genes were more up-regulated in the treated samples than in the controls (Fig. 4). For the green-yellow module, 26 out of the 301 genes were selected and divided into two submodules. The first submodule comprised Kelch-like protein 18 (Vitvi08g01787), Acidic endochitinase (CHIB1) (Vitvi16g01979), UDP-glucose glucosyltransferase (Vitvi12g01697), 2 Anthocyanidin glucosyltransferases (Vitvi03g01265, Vitvi18g02014), Desacetoxyvindoline 4-hydroxylase (Vitvi05g01929), WRKY 65 (Vitvi10g00618), PR4 (Vitvi14g00488), C2 domain-containing protein (Vitvi16g01928), Phytosulfokines PSK2 (Vitvi03g00712), ABC transporter G member 22 (Vitvi06g01950), Rubber elongation factor (Vitvi14g00167) and an unknown protein (Vitvi13g02053), Methylenetetrahydrofolate reductase-like (Vitvi05g01704) and GNK2 domain containing protein/cysteine-rich repeat secretory protein (Vitvi07g01133). A low expression of the module’s genes was observed in the control compared to a strong up-regulation followed by decreasing up-regulation in the treated samples (Fig. 4). The second submodule was composed of 8 stilbene synthases (Vitvi16g01453, Vitvi16g01461, Vitvi16g00991, Vitvi16g01457, Vitvi16g01470, Vitvi16g01472, Vitvi16g00993, Vitvi16g01469, Vitvi07g00598), 1 Phenylalanine Ammonia Lyase (Vitvi16g00057), MYB14 (Vitvi07g00598), a 4-coumarate-CoA ligase 2 (Vitvi11g01257). An up-regulation was observed at 72 hpi in the control, whereas in all the treated samples a strong down-regulation was observed at 24 h and 72 h (Fig. 4). This highlights that a large part of the stilbene biosynthesis pathway is repressed in the treated samples compared to the control. For the turquoise module, 24 out of 2901 were selected and divided into three submodules. The first submodule contained only one gene: a Senescence-associated protein (Vitvi15g00507). This gene was slightly up-regulated in the control, slightly down-regulated in P and strongly down-regulated in PT and T (Fig. 4). The second submodule was composed of 8 genes comprising a L-type lectin protein kinase (Vitvi08g01744), a S-receptor kinase (Vitvi05g00482), a calcium-transporting ATPase 13 (Vitvi10g00269), a heat shock transcription factor 4 (Vitvi07g01749), an ankyrin repeat (Vitvi04g01848), Glutathione S-transferase 25 GSTU7 (Vitvi17g01382), Ribosomal protein L36a/L44 (Vitvi01g00163) and a Respiratory burst oxidase protein F (Vitvi02g00048). A slight up-regulation of the latter genes was observed in the controls at 72 h and a strong up-regulation in the treated samples from 24 to 48 h, followed by a decrease in up-regulation at 72 h (Fig. 4). The third submodule was composed of 15 genes with Endo-1,4-beta-glucanase korrigan (Vitvi07g02990), CYP94A1 (Vitvi07g00012), Aspartate aminotransferase P1 (Vitvi04g00328), Chitinase A (Vitvi11g00634), Aconitate hydratase (Vitvi12g00505), RPM1 (Vitvi19g00795), MPK13 (Vitvi06g00365), C2 domain-containing protein (Vitvi16g01929), Ser/Thr protein kinase (Vitvi16g00474), S-locus lectin protein kinase (Vitvi10g02322), Boron transporter-like protein 4 (Vitvi09g00500), Nodulation protein (Vitvi07g01773), a putative Calcium-transporting ATPase (Vitvi12g00678) and two unknown proteins (Vitvi02g00161, Vitvi06g01603). These genes were up-regulated in the controls at 72 h and strongly up-regulated in the treated samples at 24 h with a subsequent decrease of expression at the following time point. For the tan module, no tighter cluster was found and no DEG were detected; however, the 3 genes exhibited lower expression in the controls and strong down-regulation in the treated samples at 24 h, followed by a down-regulation.

## Discussion

### Phenotypic data

OEOV application on leaf discs showed a significant decrease in sporulation of *P. viticola* under all treatment conditions, with the most efficient treatment conditions being the combination of preventive and post-infection application. Because OEOV treatment of *P. viticola* sporangia did not influence their overall fitness, a potential direct effect of OEOV on sporangia seems to be very small. OEOV effects on sporangia release, zoospore mobility or germination are likely also contributing factors for the direct effect on *P. viticola* sporangia but were not investigated in the present study. Several studies show the direct antimicrobial, insecticidal and antifungal activity of *origanum* essential oils in different^21,38^ plant and mammals^21,38^; thus far, only one published study showed that essential oil vapour has a phenotypic effect on *P. viticola* post-inoculation^20^. OEOV acted in a time- and duration-dependent manner. Treatment was most efficient when applied very close to inoculation and its effectiveness was correlated with the repetition of treatments (i.e., PT and P48-24 h treatments were carried out two times; Supplementary Fig. 3). From this data, it can be suggested that the duration of the plant defense enhancement is rather short. Additional experiments were performed with variations in treatment timing (− 48 h and − 24 h before inoculation). – 48 hpi exhibited a lower decrease in sporulation of *P. viticola* compared to – 24 hpi (Supplementary Fig. 3), highlighting the transient nature of plant defense enhancement. Regarding priming duration, the application of encapsulated OEO might alleviate the need for treatment repetitions due to a slow release of OEO over a period of several days, while protecting plant leaves from essential oil phytotoxicity^39^.

### Gene expression analysis

RNA-seq provides insights into the modulation of the plant transcriptome and into the metabolomic changes that prevent infection by pathogens via the biosynthesis of callose, phytoalexin and other antioxidant compounds. Standard DEG analysis only provides limited information for a large amount of generated data. The change at pathway level is obscured by the information at single gene level, making it difficult to perceive the overall response. Several tools are now available to help focus on co-expressed genes that could be considered as guilty by association and actin the same pathway. These different modules provide restricted lists of genes with valuable information. WGCNA is widely used in medical transcriptomics and has been shown to be successful in the identification of gene markers and target genes for therapy^40^. However, WGCNA is known to produce clusters with “background noise”^37^, which can be reduced by reclustering the produced module using tighter algorithms, such as *clust,* which will remove outlier genes.

### Pathogen recognition genes

Plant defense strategies consist in targeting either specific pathogen effectors or pathogen-associated molecular patterns (PAMPs), such as flagellin or chitin^41^. A myriad of receptor kinases or leucine-rich repeat receptor-like kinases (LRR- RLK) play an important role in recognizing pathogen infection. Lectin S-receptor kinases are a diverse gene family comprising three types: G-type, L-type and C-type. Their ectodomain is believed to bind carbohydrates and has been shown to be involved in plant defense^42^. While G-types are normally involved in self-incompatibility, some G-type lectins have been shown to be involved in the binding of a *Pseudomonas syringae* Hop1 effector^43^ or of exo-polysaccharide, which activates plant defenses. G-type lectin from rice has also been reported to contribute to plant defense against *Magnaporthe oryzae*^44^. Leucine-rich repeat receptor-like kinases (LRR RLK) are members of an extended gene family in land plants that are involved in various processes. Some members of the LRR RLK family have been shown to confer resistance to pathogens^45^ by binding to PAMPs or effectors and activating a signalling cascade to enhance plant defense. From the 6 receptor kinases found in the black and turquoise modules, little information can be derived, except for NB-ARC LRR (Vitvi19g00795) and G-type lectin S-receptor kinase (Vitvi10g02322). The former possesses an ortholog that confers resistance to *Pseudomonas syringae* ^46,47^, while the ortholog of the latter recognizes cleaved sphingolipid from oomycetes and activated plant defense^48^. However, we did not observe differentially expressed ceramidease that could work in tandem with this receptor. The orthologs of LRR RLK (Vitvi08g01352), L-type (Vitvi08g01744, Vitvi19g01964), G-type lectin S-receptor kinase (Vitvi05g00482), and another serine/threonine protein kinase (Vitvi16g00474) have not been characterized and do not exhibit any specific patterns upon inoculation with different pathogens. Due to the divergence between *Arabidopsis thaliana* (At) and *Vitis vinifera*, specialization events might have occurred in the *Vitis* genus. In *V. vinifera cv* Chasselas*,* the strong up-regulation of these genes at the very beginning of the infection might act either as pattern-triggered immunity or effector-triggered immunity to restrict *P. viticola* infection/internal colonization; however, their role is unclear for the moment. Further molecular characterization is required to elucidate their action.

### Signaling cascade

Pathogen recognition triggered an upregulation of genes involved in signal transduction through calcium spiking (calcium transporting ATPase Vitvi10g00269), the MAPK pathway (Vitvi06g00365 ortholog to *A. thaliana* MPK13^49^) and ROS production (Vitvi02g00048 ortholog to *A. thaliana At*RBOH F)*.* Respiratory burst oxidase protein F (RBOHF)^50,51^ is essential for activating and coordinating plant defense. RBOHF in combination with a DCD domain containing protein (Vitvi02g00161, AtNRP1) and GSTU7 (Vitvi17g01382, AtGSTU7^52^) might be key players in triggering a very localized and programmed cell death (PCD), hampering pathogen colonization of leaf tissue. In addition, the Heat shock transcription factor HSF4 (Vitvi07g01749, AtHSF4), might transmit plant defense enhancement to surrounding leaves, further limiting pathogen progression^53–55^. The restriction of carbon influx in infected leaves would reduce pathogen fitness, thus limiting secondary infection^56^. The senescence-associated protein (Vitvi15g00507), which potentially interacts with SnRK1, could modulate carbon resources allocation. This particular gene was down-regulated in treated samples compared to the inoculated control, which may indicate a restriction of carbon influx to the infected leaves, slowing down colonization or decreasing subsequent sporulation.

### Secondary metabolism

Another important strategy deployed by plants to defend themselves against pathogens, is the production of phytoalexins. In grapevine, the main class of phytoalexins are stilbenoids (resveratrol, pterostilbene and viniferines)^57–59^. These phytoalexins accumulate in the leaves of resistant cultivars (Solaris, Rpv3-1, *Muscadinia rotundifolia*) during infection by *P. viticola* and *E. necator*^60^*,* and are therefore thought to be directly involved in resistance to both these fungal pathogens^17,61^. Parts of the stilbene biosynthesis pathway have been elucidated in *V. vinifera*^62^*,* with different regulators having been identified, such as MYB14^63^. Ciaffi et al.^64^ found the background STS expression in non-infected Chasselas leaves to be relatively high compared to other susceptible cultivars, equaling the STS expression of some resistant cultivars. However, upon *P. viticola* infection, STS expressions were far less modulated compared to those of resistant cultivars. The results of Ciaffi et al.^64^ suggest that the induction of stilbene biosynthesis may contribute to the base immunity of grapevine to DM.

In our mock inoculated samples, the regulation of these STS was very similar to that found in Ciaffi et al.^64^, with the exception of MYB14 regulation, which followed a slightly different pattern (data not shown). Surprisingly, out of the 48 identified STS in the *Vitis* genome, 8 genes were down-regulated in our treated samples, along with their transcription factor MYB14 andtwo upstream enzymes, which indicates a potential down-regulation of this pathway. It can be hypothesized that, due to the strong down-regulation of a part of the stilbene pathway, the biosynthesis and accumulation of stilbenes is quite low and the presence of stilbenes might not have been responsible for the increased resistance to *P. viticola* in our study. An analysis of the accumulation of secondary metabolites could elucidate the potential rewiring of the phenylpropanoid pathway.

Overall, the findings suggest that the defense mechanism in our study is multi-layered, involving the recognition of *P. viticola* by different receptor families and subsequent signaling pathways that lead to a localized PCD and limited leaf colonization by the pathogen. Further studies are needed to fully elucidate the exact mechanism of this pathway.

### Meta-analysis of public RNA-seq data

While numerous resistant cultivars exhibited a strong accumulation of stilbenoids during the infection by *P. viticola*, their transcriptome also highlights the regulation of other pathways involved in plant defense, such as hypersensitive response (HR) and PCD, as has been observed in cv. Bianca^30^. Other cultivars rely exclusively on the up-regulation of various receptors and MAPKs, such as cv. Mgaloblishvilii^30^, resulting potentially in an increase in PCD or HR processes, thus restricting the propagation of *P. viticola* very early on, and subsequently hindering the overall quantity of sporangia.

In our study, the induced resistance to *P. viticola* seems to be independent of increased stilbene synthesis. To identify common resistant genes that could help to better understand the regulation of plant resistance to *P. viticola,* we searched the NCBI database for RNA-seq studies on DM conducted on resistant cultivars; we found two studies with similar designs on three different cultivars: Bianca bearing the Rpv3 locus, cv. Mgaloblishvilii with no QTL of resistance identified thus far, and a *V. amurensis* hybrid variety bearing the Rpv12 locus in an Rpv3 background without the Rpv3 QTL^30,65^. To minimize variation due to the analytic methodology used in the different transcriptomic studies^30,65^, we re-analyzed the datasets using our in-house pipeline.

For each RNA-seq data, we built a de novo transcriptome assembly and removed every gene that mapped on the PN40024 reference genome and the PV221 reference genome. We then performed the alignment of reads on the combined assembly of PN40024 (*V. vinifera*), P221 (*P. viticola*) and de novo transcriptome, obtaining the raw counts data.

While in our re-analysis a slightly lower number of DEGs was detected overall, our pipeline produced comparable results to the published data.

From these data, we built a consensus gene co-expression network (see “Materials and methods”). We obtained 188 genes from the consensus gene co-expression network using the Chitarrini, et al.^65^ and Toffolatti et al*.*^30^ study with an MM above the 0.8 threshold. Out of the 188 genes, 140 genes were from the *P. viticola* genome and 48 genes from the *V. vinifera* genome*.* Of the 48 *Vitis* genes, 20 were different types of receptor-like genes (Disease resistance, LRR RLK, Lys Motif-Type Receptor-Like Kinase, R protein, LRK, RPM1 and S-receptor kinase) (Supplementary Table 2). Very few common DEGs with an absolute log fold change above 1 could be retrieved from these combined studies. We thus focused on genes that were DE at the 24 h time point to explore the early plant response. Thirty-nine DEGs were retrieved with only four *Vitis* DEGs from the three cultivars: a Ser/Thr receptor-like kinase1 (Vitvi00g02226), one Receptor-like protein (Vitvi18g03246) and two G-type lectin S-receptor-like serine/threonine-protein kinase (Vitvi19g01957, Vitvi19g01959). As previously observed in our RNA-seq analysis, the majority of these genes belong to the receptor kinase class, further enforcing the essential role of these genes in the resistance process against *P. viticola* across different cultivars*.*

As regards the pathogen, due to a strong focus on secreted protein of the community, the rest of the genome lacks in-depth functional annotation; however, several heat shock-related proteins were retrieved, along with stress-related proteins and ubiquitin-related proteins (Supplementary Table 2).

Our WGCNA consensus gene co-expression network identified only a small set of genes that potentially play a role in DM resistance (Supplementary Table 3). We intersected the WGCNA common gene co-expression network list (genes with an MM above 0.8) with the DEG list of each cultivar. We compared each of these lists to the *clust* DEG list that we obtained with our dataset on the Chasselas cultivar. We retrieved only one gene (Vitvi05g00482) that intersected with Chasselas and the Rpv12 hybrid. The latter is a G-type lectin S-receptor kinase, which, as mentioned above, plays a role in pathogen recognition in plant defense. Further characterization of these genes is necessary to gain a better understanding of their specific roles.

In contrast to symbiotic interactions, such as nitrogen-fixing symbiosis or arbuscular mycorrhizal symbiosis, wherein molecular mechanisms are largely well conserved^66,67^, plant-pathogen interactions are more fluctuant by nature due to their arms race dynamics, and the identification of conserved pathways or responses is more difficult, with some exceptions. Due to the diverse data sources and experimental designs, as well as differing cultivar genetic backgrounds, identifying a common pathway is a challenging task. A community effort would be necessary to establish a pan-genome that would help address this challenge and to be able to precisely identify the gene/pathways responsible for resistance phenotypes. The multiple occurrences of plant resistance should be seen as a gold mine for the creation of pyramidal resistant cultivar by combining various sources of resistant genes or pathways.

## Conclusion

The present study suggests that natural substances, such as essential oil from oregano, can prime the innate immune system of the grapevine, thereby hindering downy mildew infection. By applying essential oil during the vapor phase, commonly encountered problems, such as phytotoxicity, poor mixability in water, low rain fastness and degradation by UV light, could be circumvented. Challenges for its application in the field still exist, but rapidly developing technologies, such as encapsulations or nanoemulsion, could help overcome problems related to the application of liquid essential oils which could serve as an alternative to conventional and organic fungicides. Its preventive capacity due to its indirect effect is a major advantage in disease management.

With the data generated, we have been able to precisely identify the molecular mechanism of OEOV, which seems to be independent of the modulation of stilbene expression. The identification of resistant genes common to treated Chasselas and resistant cultivars resulted in new targets being established for breeding programs aiming at developing pyramidal resistant cultivars.

## Materials and methods

### Plants and inoculation

For all leaf disc experiments, leaves were taken from *Vitis vinifera* cv. Chasselas plants. The wood for the cv. Chasselas plant cuttings was obtained from certified vineyards of the Agroscope clonal selection program (no voucher specimen of this material has been deposited in a publicly available herbarium; appropriate permission was obtained from the land owner to enter and pick the plant wood. Plants had been grown in the greenhouse at 24/15 °C (day/night) with a 14 h photoperiod and at 70% relative humidity at Changins, Agroscope). *Plasmopara viticola* sporangia were harvested from naturally-infected leaves on vines from the experimental vineyard at Changins in Nyon, Switzerland. These *P. viticola* sporangia were recovered from the leaves by shaking them gently while suspended in water for 1 h at 50 rpm. The concentration of sporangia was determined by two independent counts on a hemocytometer.

Experimental inoculations were carried out on Chasselas leaves (2nd to 5th leaf from the apex) discs (Ø 1.1 cm) by spraying 1 mL per petri dish of a sporangia suspension containing 10^5^ sporangia/mL. Leaf discs were maintained in growth chambers (25 °C/20 °C 80% RH photoperiod 14 h) until the incubation period was over.

### Essential oil assays

#### Preventive assays

**Figure 5:**
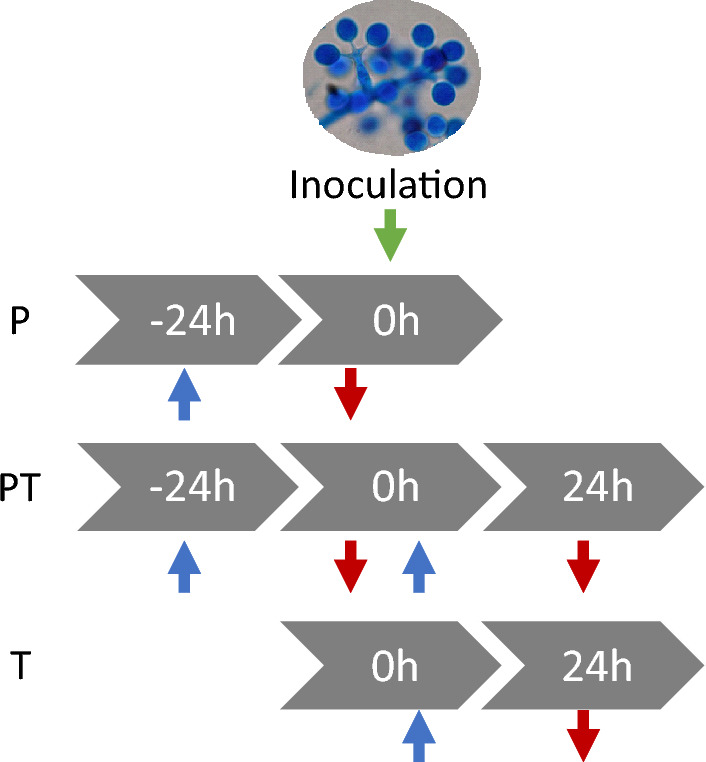
Experimental design. Blue arrow: EO treatment. Red arrow: removal of EO treatment.

An Eppendorf cap containing oregano essential oil (EO) was placed in Petri dishes (Ø 90 mm) containing 5 Chasselas leaf discs (Ø 1.1 cm) to provide oregano essential oil vapor (OEOV) at a defined time depending on the treatment conditions (see below). At T0 h the leaf discs were inoculated with 1 mL of a sporangia suspension at 10^5^ sporangia/mL, or with 1 mL of distilled water per Petri dish for the control. EO was added depending on the treatment conditions at different times, either 24 h before inoculation (primed: P) or 24 h before inoculation with a renewal of EO just after inoculation (primed and treated:PT), or just after inoculation (treated: T) (see Fig. 5). Different dosages of EO were used, ranging from 10 to 2.5 µL/L of air, to determine the most efficient dosage. The experiment was replicated three times with 3 Petri dishes per treatment conditions.

Commercially available standard EO was purchased from Compagnie des sense^®^ (France). Its composition was analysed by gas chromatography (Agilent 7890B; Capillary column: 60 m, 0.25 mm ID, 1.4 μm, Rtx^®^-1301, vector gas: hydrogen, flow 4 mL/min, injector temperature 100 °C, Gradient: 5 min at 40 °C then 3 °C/min until 240 °C, total run time 71.67 min) with Flame Ionization Detector (FID) (250 °C, Air flow 400 mL/min, H2 fuel flow 30 mL/min). The detailed composition is provided in Supplementary Table 4.

In Petri dishes (Ø 90 mm), 5 Chasselas leaf discs (Ø 1.1 cm) were sprayed with 1 mL of a sporangia suspension at 10^5^ sporangia/mL. After 10 days, an eppendorf cap containing EO (2.5 µL/L of air) was placed in the Petri dish, left for 1 h, then removed. The sporangia were harvested using the method described above and new leaf discs were inoculated with the treated sporangia at a concentration of 10^5^ sporangia/mL and the control was sprayed with distilled water. The experiment was replicated two times with 6 Petri dishes per condition.

#### Percentage of leaf covered by sporulation

After 10 days, the Petri dishes were photographed with a standard Nikon digital camera. The images were analyzed using a semi-automatic method developed by Perossotti et al. 2011 to evaluate the percentage of leaf covered by sporulation using default parameters. The leaf’s veins and edge of leaf discs were manually removed when required.

### Curative assays

#### RNA extraction and sequencing

The leaf discs were harvested at three different time points (24 hpi, 48 hpi and 72 hpi). Three Petri dishes from the same treatment conditions (15 leaf discs) were pooled to form one sample, immediately frozen in liquid nitrogen and stored at − 80 °C before extraction. RNA was extracted using Sigma Spectrum™ Plant total RNA-kit following the manufacturer’s instructions. TruSeq stranded mRNA-seq (Illumina, San Diego, CA) was used for library preparation according to the manufacturer’s instructions. Both RNA samples and final libraries were quantified with a Qubit 2.0 fluorometer (Invitrogen) and quality tested with an Agilent 2100 Bioanalyzer RNA Nano assay (Agilent Technologies, Santa Clara, CA). The libraries were then sequenced with 75 bp single-end mode with a NextSeq500 apparatus (Illumina). All sequencing data is available on NCIBI under the following link in the form of fastq files: https://dataview.ncbi.nlm.nih.gov/object/PRJNA879269?reviewer=n107n11gult5rlom1ik0so6ofi&archive=sra.

### De novo assembly

To recover transcripts, which are absent in the PN44024 reference genome, de novo transcriptome assembly was performed. Raw reads were processed using in-house Python script following the guideline written by (https://informatics.fas.harvard.edu/best-practices-for-de-novo-transcriptome-assembly-with-trinity.html). Uncorrected paired reads were tagged with rCorrector, then removed with a python script FilterUncorrectabledPEfastq.py. These reads were then trimmed with Trim Galore! and finally aligned with bowtie2 on a concatenated database containing the SILVA database, the *V. vinifera* reference genome PN40024 12X v2 and the *P.viticola* reference genome PV221 (https://github.com/colarigo/WGCNA/blob/main/de_novo_trans_pipeline.py). Only paired reads unaligned to the concatenated database were retrieved. Trinity followed by Transdecoder were used to assemble the transcripts, and retrieve the longest ORF and the most representative transcripts (https://github.com/colarigo/WGCNA/blob/main/trinity_pipe.py). Interproscan was used to provide basic annotation of the newly assembled transcripts (https://github.com/colarigo/WGCNA/blob/main/interproscan_launcher.py).

### RNA-seq analysis

Raw reads were processed by in-house Python script using trimmomatic 0.36, hisat2 aligned on a concatenated database containing the *V.vinifera* reference genome PN40024 12X V2, the *P.viticola* reference genome PV221, and the partial de novo transcriptome assembly produced in the precedent step, samtools (view, sort by name, fixmate, sort by order, markdup, sort by name), FeatureCounts which calculated counts using a concatenated gff containing PN40024, PV221, partial de novo transcriptome assembly (https://github.com/colarigo/WGCNA/blob/main/transcriptomic_pipeline.py). The calculation of differentially expressed genes was performed with DESeq2. Venn diagram was performed on DEG genes using an online tool (https://bioinformatics.psb.ugent.be/webtools/Venn/).

### WGCNA analysis

The resulting FeatureCounts matrices for the inoculated control, P, PT, and T conditions were processed using Deseq2 and ComBat-seq packages (https://github.com/colarigo/WGCNA/blob/main/WCGNA_preprocessing_ComBat_seq_vst_table.R). An ACP analysis was performed using R with on each of the resulting matrices?

A WGCNA package was used to cluster samples and remove outliers. softPower was calculated through scale free topology with a threshold of > 0.9 for sqt.R and > 0.9 for truncated sqt.R, and < 0 for slope. Modules were calculated with *blockwiseModules* function and deepsplit 0, above calculated softPower. Module trait correlation was calculated with the *cor* function using MEs and median of sporulation for each of the treatment conditions. Only the four highest correlated modules to trait were subjected to further analysis. Module membership was calculated for each gene in each module and only genes with an MM > 0.8 for the four highest correlated modules were conserved (https://github.com/colarigo/WGCNA/blob/main/WGCNA_network_building_exploration.R). Expression of genes with MM > 0.8 was explored using R.

### GO analysis

Genes belonging to the four highest correlated modules retrieved from the above WGCNA analysis (MM > 0.8) were tested for GO enrichment using the *goseq* package. Missing GO terms were retrieved through quickGO API (https://github.com/colarigo/WGCNA/blob/main/go_seq_modules.R).

### clust analysis

Due to WGCNA noisy module clustering, *clust* (https://github.com/BaselAbujamous/clust) was used to properly redefine modules and remove any outlier genes.

Previous genes with MM > 0.8 for the highest correlated modules were used and DESeq2 matrices were restricted to this selection of genes for each module. *clust* was performed with default parameters except for the normalization parameter (-n4). (https://github.com/colarigo/WGCNA/blob/main/clust_analysis_for_multi_module.py).

### Public data analysis

NCBI was screened for studies with similar experimental designs and sequencing platforms; two studies were retrieved from the *SRA* repository^30,65^. These studies include resistant (Bianca, Mgaloblishvilii and hybrid with *V. amurensis*) andsensitive (Pinot Noir) cultivars. Raw reads were analysed as previously described with some modifications. WGCNA analysis was done using *blockwiseconsensusModules* function. Module membership was calculated using *consensusKME* function. Genes with MM > . 8 from the highest correlated modules were conserved. Resistant cultivars data were analysed together to retrieve potential common resistant genes. Sensible cultivars (Pinot Noir data from Toffolatti et al., 2018 and Chasselas control) were analysed together to retrieve susceptible genes (https://github.com/colarigo/WGCNA/blob/main/WGCNA_consensus_network_building_exploration.R).

### DEG analysis

To identify the genes that were differentially regulated and strongly expressed in the treated and the inoculated control, the genes were filtered with a p-value of < 0.05 and an absolute log value above 1.

### Candidate genes

To identify genes common to resistant cultivars and EO-treated Chasselas, an intersection between genes MM > 0.8 resistant cultivars and MM > 0.8 EO-treated Chasselas was applied. The intersected genes that were also present in the susceptible genes list were removed.

### Research involving plants

We confirm that all methods were carried out in accordance with relevant guidelines in the method section.

### Supplementary Information

Supplementary Information.

Supplementary Table 1.

Supplementary Table 2.

Supplementary Table 3.

Supplementary Table 4.

## Data Availability

All sequencing data in the form of fastq files is available on NCBI (https://www.ncbi.nlm.nih.gov/) under the following bioproject number PRJNA879269.

## References

[1] International Organisation of Vine and Wine, O. State of the world vine and wine sector 2022 (2022).

[2] Asghar U, Malik MF (2016). Pesticide exposure and human health: A review. J. Ecosyst. Ecogr..

[3] Rienth M (2021). Modifications of grapevine berry composition induced by main viral and fungal pathogens in a climate change scenario. Front. Plant Sci..

[4] Koledenkova K (2022). *Plasmopara viticola* the causal agent of downy mildew of grapevine: From its taxonomy to disease management. Front. Microbiol..

[5] Droz B (2021). Copper content and export in European vineyard soils influenced by climate and soil properties. Environ. Sci. Technol..

[6] Massi F, Torriani SFF, Borghi L, Toffolatti SL (2021). Fungicide resistance evolution and detection in plant pathogens: *Plasmopara viticola* as a case study. Microorganisms.

[7] Dagostin S, Formolo T, Giovannini O, Pertot I, Schmitt A (2010). *Salvia officinalis* extract can protect grapevine against *Plasmopara viticola*. Plant Dis..

[8] Singh RK (2022). Viewpoint of chitosan application in grapevine for abiotic stress/disease management towards more resilient viticulture practices. Agriculture.

[9] Dagostin S, Schärer H-J, Pertot I, Tamm L (2011). Are there alternatives to copper for controlling grapevine downy mildew in organic viticulture?. Crop Prot..

[10] Nerva L, Sandrini M, Gambino G, Chitarra W (2020). Double-stranded RNAs (dsRNAs) as a sustainable tool against gray mold (*Botrytis cinerea*) in grapevine: Effectiveness of different application methods in an open-air environment. Biomolecules.

[11] Gauthier A (2014). The sulfated laminarin triggers a stress transcriptome before priming the SA- and ROS-dependent defenses during grapevine's induced resistance against *Plasmopara viticola*. PLoS ONE.

[12] Héloir M-C (2018). Assessment of the impact of PS3-induced resistance to downy mildew on grapevine physiology. Plant Physiol. Biochem..

[13] Burdziej A (2021). Three types of elicitors induce grapevine resistance against downy mildew via common and specific immune responses. J. Agric. Food Chem..

[14] Colombo M (2020). NoPv1: A synthetic antimicrobial peptide aptamer targeting the causal agents of grapevine downy mildew and potato late blight. Sci. Rep..

[15] Hamiduzzaman MM, Jakab G, Barnavon L, Neuhaus JM, Mauch-Mani B (2005). beta-Aminobutyric acid-induced resistance against downy mildew in grapevine acts through the potentiation of callose formation and jasmonic acid signaling. Mol. Plant Microbe Interact. MPMI.

[16] Wang Y (2022). Kaolin particle film limits grapevine downy mildew epidemic under open-field conditions and stimulates the plant defence response. Aust. J. Grape Wine Res..

[17] Mijailovic N, Nesler A, Perazzolli M, Aziz A, Essaïd AB (2022). Foliar application of a tagatose-based product reduces downy mildew symptoms through induction of grapevine resistance and anti-oomycete action. Agronomy.

[18] Perazzolli M (2012). Downy mildew resistance induced by Trichoderma harzianum T39 in susceptible grapevines partially mimics transcriptional changes of resistant genotypes. BMC Genom..

[19] De Miccolis Angelini RM (2019). Global transcriptome analysis and differentially expressed genes in grapevine after application of the yeast-derived defense inducer cerevisane. Pest Manag. Sci..

[20] Fialho R, Papa M, Panosso A, Cassiolato A (2017). Fungitoxicity of essential oils on plasmapora viticola, causal agent of grapevine downy mildew. Rev. Bras. Frutic..

[21] Kakouri E (2022). *Origanum majorana* essential oil; A review of its chemical profile and pesticide activity. Life.

[22] Marrelli M, Statti GA, Conforti F (2018). Origanum spp.: An update of their chemical and biological profiles. Phytochem. Rev..

[23] Rienth M, Crovadore M, Ghaffari S, Lefort F (2019). Oregano essential oil vapour prevents *Plasmopora viticola* infection in grapevine (*Vitis vinifera*) by triggering autoimmune metabolic pathway. PLoS ONE.

[24] Burggraf A, Rienth M (2020). *Origanum vulgare* essential oil vapour impedes Botrytis cinerea development on grapevine (*Vitis vinifera*) fruit. Phytopathol. Mediterr..

[25] Mauch-Mani B, Baccelli I, Luna E, Flors V (2017). Defense priming: An adaptive part of induced resistance. Annu. Rev. Plant Biol..

[26] Burdziej A (2019). Impact of different elicitors on grapevine leaf metabolism monitored by 1H NMR spectroscopy. Metabolomics.

[27] Piasecka A, Jedrzejczak-Rey N, Bednarek P (2015). Secondary metabolites in plant innate immunity: Conserved function of divergent chemicals. New Phytol.

[28] Polesani M (2010). General and species-specific transcriptional responses to downy mildew infection in a susceptible (*Vitis vinifera*) and a resistant (*V. riparia*) grapevine species. BMC Genom..

[29] Fröbel S, Dudenhöffer J, Töpfer R, Zyprian E (2019). Transcriptome analysis of early downy mildew (*Plasmopara viticola*) defense in grapevines carrying the Asian resistance locus Rpv10. Euphytica.

[30] Toffolatti SL (2018). Unique resistance traits against downy mildew from the center of origin of grapevine (*Vitis vinifera*). Sci. Rep..

[31] Toffolatti SL (2020). Novel aspects on the interaction between grapevine and *Plasmopara viticola*: Dual-RNA-Seq analysis highlights gene expression dynamics in the pathogen and the plant during the battle for infection. Genes.

[32] Eisenmann B (2019). Rpv3-1 mediated resistance to grapevine downy mildew is associated with specific host transcriptional responses and the accumulation of stilbenes. BMC Plant Biol..

[33] Brilli M (2018). A multi-omics study of the grapevine-downy mildew (*Plasmopara viticola*) pathosystem unveils a complex protein coding- and noncoding-based arms race during infection. Sci. Rep..

[34] OIV. *Descriptor List for Grape Varieties and Vitis Species* (Office International de la Vigne et du Vin, 2009).

[35] Peressotti E, Duchêne E, Merdinoglu D, Mestre P (2011). A semi-automatic non-destructive method to quantify grapevine downy mildew sporulation. J. Microbiol. Methods.

[36] Kim Khiook IL (2013). Image analysis methods for assessment of H_2_O_2_ production and *Plasmopara viticola* development in grapevine leaves: Application to the evaluation of resistance to downy mildew. J. Microbiol. Methods.

[37] Abu-Jamous B, Kelly S (2018). Clust: Automatic extraction of optimal co-expressed gene clusters from gene expression data. Genome Biol..

[38] Sharifi-Rad J (2021). Phytochemical constituents, biological activities, and health-promoting effects of the *Melissa officinalis*. Oxid. Med. Cell. Longev..

[39] Maes C, Bouquillon S, Fauconnier M-L (2019). Encapsulation of essential oils for the development of biosourced pesticides with controlled release: A review. Molecules (Basel Switzerland).

[40] Langfelder P, Horvath S (2008). WGCNA: An R package for weighted correlation network analysis. BMC Bioinform..

[41] Jones JD, Dangl JL (2006). The plant immune system. Nature.

[42] Sun Y, Qiao Z, Muchero W, Chen J-G (2020). Lectin receptor-like kinases: The sensor and mediator at the plant cell surface. Front. Plant Sci..

[43] Ranf S (2015). A lectin S-domain receptor kinase mediates lipopolysaccharide sensing in *Arabidopsis thaliana*. Nat. Immunol..

[44] Chen X (2006). A B-lectin receptor kinase gene conferring rice blast resistance. Plant J..

[45] Liebrand TWH, van den Burg HA, Joosten MHAJ (2014). Two for all: receptor-associated kinases SOBIR1 and BAK1. Trends Plant Sci..

[46] Yoon M, Middleditch MJ, Rikkerink EHA (2022). A conserved glutamate residue in RPM1-INTERACTING PROTEIN4 is ADP-ribosylated by the Pseudomonas effector AvrRpm2 to activate RPM1-mediated plant resistance. Plant Cell.

[47] Boyes DC, Nam J, Dangl JL (1998). The *Arabidopsis thaliana*
*RPM1* disease resistance gene product is a peripheral plasma membrane protein that is degraded coincident with the hypersensitive response. Proc. Natl. Acad. Sci..

[48] Kato H (2022). Recognition of pathogen-derived sphingolipids in Arabidopsis. Science.

[49] Nitta Y, Ding P, Zhang Y (2014). Identification of additional MAP kinases activated upon PAMP treatment. Plant Signal Behav..

[50] Marcec MJ, Tanaka K (2022). Crosstalk between calcium and ROS signaling during Flg22-triggered immune response in Arabidopsis leaves. Plants.

[51] Fichman Y (2023). Phytochrome B regulates reactive oxygen signaling during abiotic and biotic stress in plants. New Phytol..

[52] Sappl PG, Oñate-Sánchez L, Singh KB, Millar AH (2004). Proteomic analysis of glutathione S-transferases of *Arabidopsis thaliana* reveals differential salicylic acid-induced expression of the plant-specific phi and tau classes. Plant Mol. Biol..

[53] Pick T, Jaskiewicz M, Peterhänsel C, Conrath U (2012). Heat shock factor HsfB1 primes gene transcription and systemic acquired resistance in Arabidopsis. Plant Physiol..

[54] Xu G (2017). uORF-mediated translation allows engineered plant disease resistance without fitness costs. Nature.

[55] Kumar M (2009). Heat shock factors HsfB1 and HsfB2b are involved in the regulation of Pdf1.2 expression and pathogen resistance in Arabidopsis. Mol. Plant.

[56] Schwachtje J (2006). SNF1-related kinases allow plants to tolerate herbivory by allocating carbon to roots. Proc. Natl. Acad. Sci..

[57] Gindro, K. *et al.* In *Plant Defence: Biological Control* (eds. Mérillon, J. M. & Ramawat, K. G.) 25–54 (Springer Netherlands, 2012).

[58] Latouche G, Bellow S, Poutaraud A, Meyer S, Cerovic ZG (2013). Influence of constitutive phenolic compounds on the response of grapevine (*Vitis vinifera* L.) leaves to infection by *Plasmopara viticola*. Planta.

[59] Pezet R (2003). delta-viniferin, a resveratrol dehydrodimer: One of the major stilbenes synthesized by grapevine leaves. J. Agric. Food Chem..

[60] Viret O, Spring J-L, Gindro K (2018). Stilbenes: Biomarkers of grapevine resistance to fungal diseases. OENO One.

[61] Schnee S, Viret O, Gindro K (2008). Role of stilbenes in the resistance of grapevine to powdery mildew. Physiol. Mol. Plant Pathol..

[62] Vannozzi A (2018). Combinatorial regulation of stilbene synthase genes by WRKY and MYB transcription factors in grapevine (*Vitis vinifera* L.). Plant Cell Physiol..

[63] Holl J (2013). The R2R3-MYB transcription factors MYB14 and MYB15 regulate stilbene biosynthesis in *Vitis vinifera*. Plant Cell.

[64] Ciaffi M (2019). Transcriptional regulation of stilbene synthases in grapevine germplasm differentially susceptible to downy mildew. BMC Plant Biol..

[65] Chitarrini G (2020). Two-omics data revealed commonalities and differences between Rpv12- and Rpv3-mediated resistance in grapevine. Sci. Rep..

[66] Rich MK (2021). Lipid exchanges drove the evolution of mutualism during plant terrestrialization. Science.

[67] Cathebras C (2022). A novel cis element enabled bacterial uptake by plant cells. bioRxiv.

