# Supplementary material for: Unravelling molecular mechanisms involved in resistance priming against downy mildew (Plasmopara viticola) in grapevine (Vitis vinifera L.)
Source: Sci Rep. 2023 Sep 6;13:14664. doi: 10.1038/s41598-023-41981-x (PMC10482922; doi:10.1038/s41598-023-41981-x)
Supplement: Supplementary file 1 — Supplementary Information. [file 41598_2023_41981_MOESM1_ESM.docx]

**Supplementary Information legends**

**Supplementary Table 1 :**

List of differentially expressed genes per top modules

**Supplementary Table 2 :**

List of differentially expressed genes per top modules in public RNA-seq data

**Supplementary Table 3 :**

List of genes identified by WGCNA

**Supplementary Table 4 :**

Composition of essential oil.

**Supplementary tables:**

**Supplementary Figure 1:**

Dose dependent effect of Oregano essential oil vapor

**Supplementary Figure 2:**

Genes clustering in response to OEOV and *P.viticola.* A/ Dendrogram clustering genes per module and gene significance. B/ Matrix of module and percentage of sporulation correlation.

**Supplementary Figure. 3:**

Time dependent effect of Oregano essential oil vapor


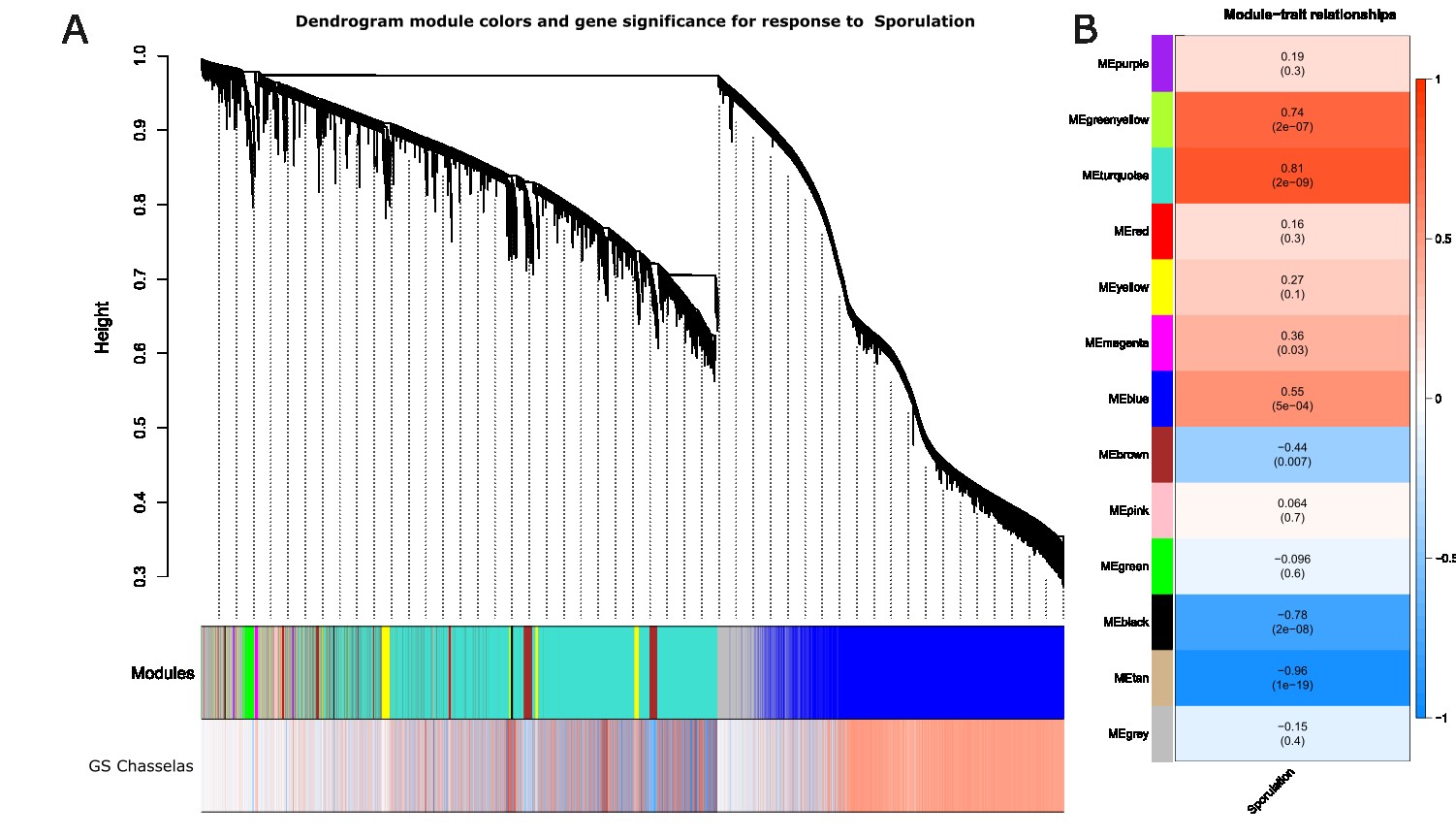


**Supplementary Figure 1:**


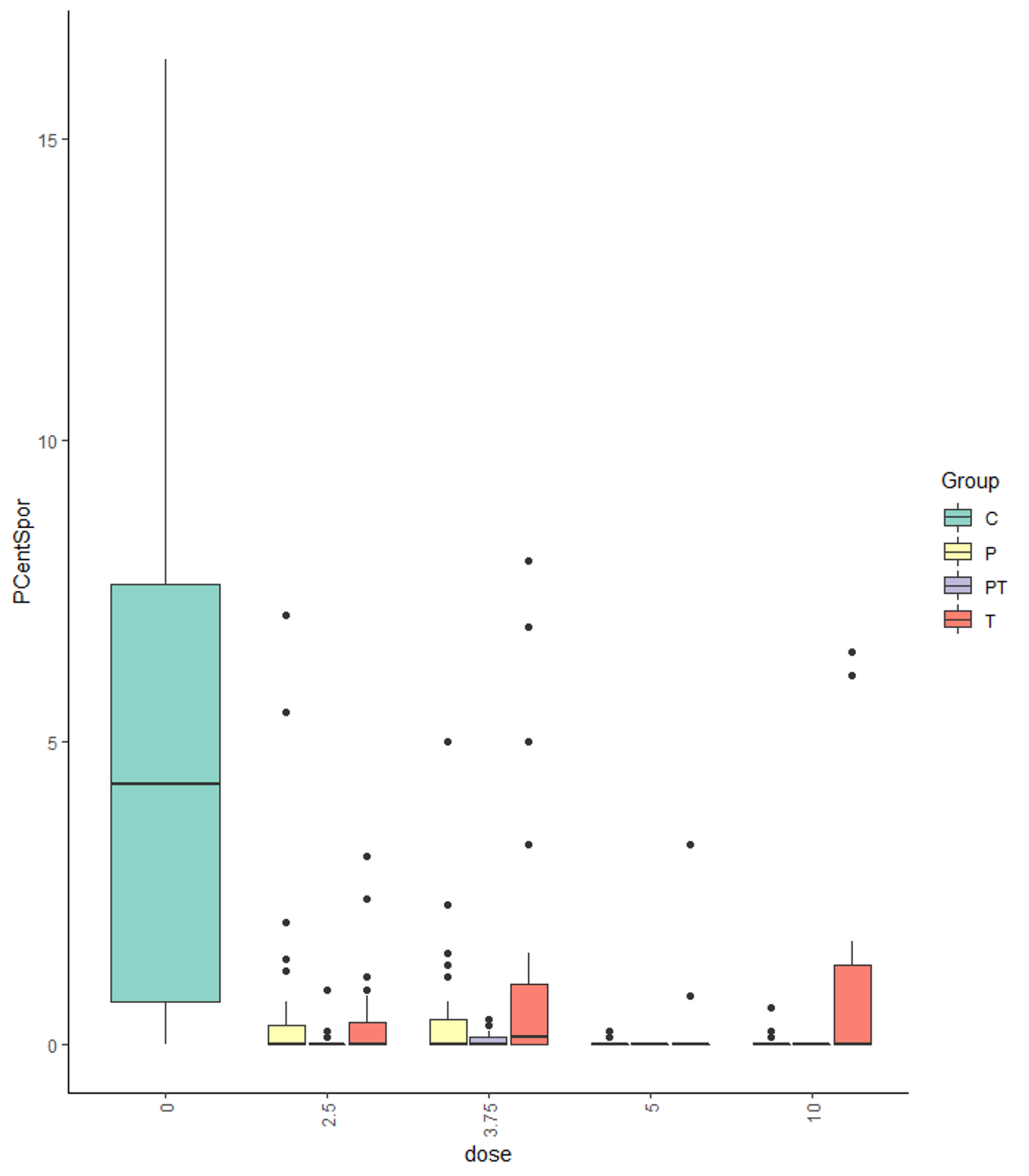


**Supplementary Figure 2:**


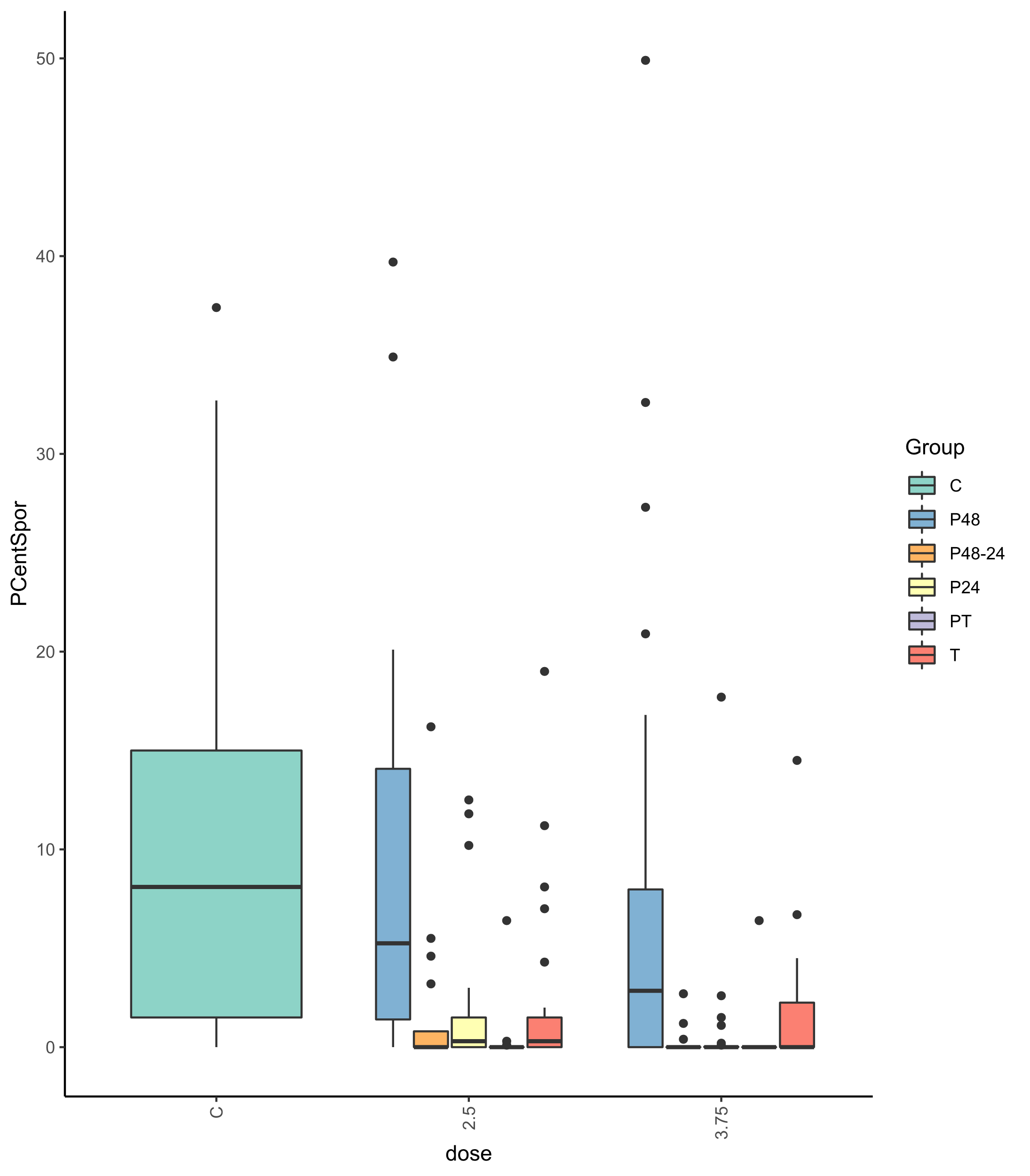


**Supplementary Figure. 3 :**
